# Factors associated with anxiety during the first two years of the COVID-19 pandemic in the United States: An analysis of the COVID-19 Citizen Science study

**DOI:** 10.1371/journal.pone.0297922

**Published:** 2024-02-06

**Authors:** Aaron E. Cozen, Thomas Carton, Rita Hamad, John Kornak, Madelaine Faulkner Modrow, Noah D. Peyser, Soo Park, Jaime H. Orozco, Matthew Brandner, Emily C. O’Brien, Djeneba Audrey Djibo, Cheryl N. McMahill-Walraven, Carmen R. Isasi, Alexis L. Beatty, Jeffrey E. Olgin, Gregory M. Marcus, Mark J. Pletcher

**Affiliations:** 1 Department of Epidemiology and Biostatistics, University of California, San Francisco, San Francisco, CA, United States of America; 2 Louisiana Public Health Institute, New Orleans, LA, United States of America; 3 Dept of Social and Behavioral Sciences, Harvard School of Public Health, Boston, MA, United States of America; 4 Division of Cardiology, University of California, San Francisco, San Francisco, CA, United States of America; 5 Department of Family and Community Medicine, Philip R. Lee Institute for Health Policy Studies, University of California, San Francisco, San Francisco, CA, United States of America; 6 Duke Clinical Research Institute, Durham, NC, United States of America; 7 CVS Health Clinical Trial Services, Blue Bell, PA, United States of America; 8 Department of Epidemiology, Albert Einstein College of Medicine, The Bronx, NY, United States of America; Iranian Institute for Health Sciences Research, ISLAMIC REPUBLIC OF IRAN

## Abstract

COVID-19 increased the prevalence of clinically significant anxiety in the United States. To investigate contributing factors we analyzed anxiety, reported online via monthly Generalized Anxiety Disorders-7 (GAD-7) surveys between April 2020 and May 2022, in association with self-reported worry about the health effects of COVID-19, economic difficulty, personal COVID-19 experience, and subjective social status. 333,292 anxiety surveys from 50,172 participants (82% non-Hispanic white; 73% female; median age 55, IQR 42–66) showed high levels of anxiety, especially early in the pandemic. Anxiety scores showed strong independent associations with worry about the health effects of COVID-19 for oneself or family members (GAD-7 score +3.28 for highest vs. lowest category; 95% confidence interval: 3.24, 3.33; p<0.0001 for trend) and with difficulty paying for basic living expenses (+2.06; 1.97, 2.15, p<0.0001) in multivariable regression models after adjusting for demographic characteristics, COVID-19 case rates and death rates, and personal COVID-19 experience. High levels of COVID-19 health worry and economic stress were each more common among participants reporting lower subjective social status, and median anxiety scores for those experiencing both were in the range considered indicative of moderate to severe clinical anxiety disorders. In summary, health worry and economic difficulty both contributed to high rates of anxiety during the first two years of the COVID-19 pandemic in the US, especially in disadvantaged socioeconomic groups. Programs to address both health concerns and economic insecurity in vulnerable populations could help mitigate pandemic impacts on anxiety and mental health.

## Introduction

Generalized anxiety and other mental health disorders were leading contributors to morbidity in the US and worldwide in the 20-year period preceding COVID-19 [1, 2]. The prevalence of clinically significant anxiety increased by 25–30% or more in the US and many other countries during the first year of the pandemic [1, 3, 4]. While SARS-CoV-2 infection itself may cause anxiety [5, 6], it is likely that pandemic-related social and economic factors including worry about the health effects of COVID-19, and financial hardship resulting from or exacerbated by pandemic economic disruptions have also contributed to increases in anxiety [4, 7, 8]. Understanding which factors are more important, for whom, and how they interact to cause anxiety may help policymakers and healthcare providers prepare for and respond to the ongoing effects of COVID-19, and future pandemics.

More than one million Americans have died from COVID-19, with especially high rates of morbidity and mortality among older adults, marginalized racial and ethnic groups, people with elevated risks of infection at work or at home, and people with less access to healthcare and vaccines [9–13]. The economic impacts of COVID-19 and pandemic-related restrictions have also been extensive and unequal. Contributing factors included high rates of unemployment, higher risk of COVID-19 exposure at work among workers with lower levels of education, loss of income for workers without paid sick leave, and reduced labor force participation for family care-givers following COVID-19 illness [8, 10, 14–16]. Understanding how these factors contribute to anxiety and other mental health conditions that have worsened during the pandemic is important for clinicians caring for patients with anxiety, and for policymakers seeking to balance pandemic risk reduction against economic considerations.

To investigate factors associated with anxiety over the first two years of the pandemic, we analyzed survey responses from the COVID-19 Citizen Science (CCS) study [17], an ongoing, entirely digital, longitudinal cohort study which has enrolled over 100,000 participants since its March 26, 2020 launch. The large cohort and longitudinal study design with repeated measures of anxiety and depression, along with the wide range of information collected on demographics, household characteristics, medical conditions, and occupational activities, present a unique opportunity to investigate factors associated with changes in mental health over the course of the pandemic. Given well-documented disparities in the health and economic impacts of the pandemic in the United States, we hypothesized that worry about the health effects of COVID-19 and economic distress were important contributors to anxiety, and that both factors were particularly important for socioeconomically vulnerable groups.

## Methods

### CCS study overview

The COVID-19 Citizen Science study (CCS) is a longitudinal cohort study launched March 26, 2020 on the Eureka Research Platform [18] to study COVID-19 testing, outcomes and patient-reported impact of the pandemic. Methods for the CCS study were described previously [17]. The recruitment period for this study of anxiety started March 26, 2020 and ended May 4, 2022; recruitment for the CCS study is ongoing with no prespecified enrollment targets or end dates for enrollment or completion. Participants were recruited by emailing participants in other Eureka platform studies, by press and word of mouth (including social media), and through partner programs and collaborating health systems [17, 19]. Initially, adults 18 or older were eligible if they had an iOS or Android smartphone with a valid cell phone number and agreed to participate in English; beginning January 2021 a parallel website was launched to enable recruitment without a smartphone. After receiving information about the study and providing electronic consent to participate, participants were asked to complete a baseline survey about demographics, medical conditions, medications, and household characteristics, followed by weekly and monthly surveys about activities, health, attitudes and experience during the pandemic. All surveys were administered in English through the Eureka app or website. Participants were surveyed about symptoms of anxiety on a monthly basis beginning April 22, 2020. Engagement with recurring surveys was encouraged using push notifications, SMS text and email messages with a link to the Eureka app or website. The study was reviewed and approved by the University of California, San Francisco Institutional Review Board (17–21879).

### Study sample

We analyzed anxiety survey results returned between April 22, 2020 and May 4, 2022 from participants with complete baseline demographic characteristics including subjective social status [20], and a zip code identifiable in US government databases that was linkable with county-level COVID-19 case and death rates obtained from the New York Times [21]. Anxiety responses from these participants were included if we also had complete concurrent information regarding economic difficulty, daily occupational activities, personal COVID-19 experience including results of tests for active COVID-19 infection, and worry about the health impacts of COVID-19 in recurring surveys. Recurring surveys were delivered on a weekly or monthly basis, balancing participant burden and timeliness of time-varying factors.

### Measurements

Our primary outcome–anxiety–was measured in monthly surveys using a seven-item questionnaire for Generalized Anxiety Disorder (GAD-7), a validated survey with high reliability as a screening tool [22, 23]. Responses to each item in the GAD-7 survey are scored as integer values between 0–3 according to the frequency of symptoms during the preceding two weeks (“Not at all”, “Several days”, “More than half the days”, or “Nearly every day”), and the sum of all seven items produces a combined score between 0–21 [22, 23] (S1 Table). Incomplete GAD-7 surveys missing responses to any of the seven questionnaire items were removed from analysis.

We focused on associations with two potential primary drivers of anxiety: worry about the health effects of COVID-19, and economic difficulty. In each case, we analyzed concurrent responses provided on the same day as anxiety survey responses. Worry about health impacts of COVID-19 was measured by a single survey question, delivered weekly: “Over the past WEEK, how worried have you been that the health of you or your loved ones will be affected by COVID-19?”. Self-reported economic stress was recorded in monthly survey responses to two separate questions: “How hard is it for you (and your family) to pay for the very basics like food, rent or mortgage, heating, etc. over the past 30 days?” and “Did you have difficulty making ends meet over the past 30 days?”. We used the first question about difficulty paying for basic living expenses in our primary analysis of economic stress, and the second question about making ends meet in a supplemental analysis.

Additional measurements were used in analyses. MacArthur scale subjective social status values between 1 (lowest) and 10 (highest) [20], age, gender, race/ethnicity, medical conditions, education, employment field, living with school-aged children (grades K-12), and country of residence and zip code were recorded in baseline surveys. Income change, working from home and primary daily activities were recorded monthly. Daily county-level COVID-19 case and death rates from The New York Times (calculated as the trailing seven-day average per 100,000) were matched to corresponding anxiety survey responses using zip code-county crosswalk files from the Department of Housing and Urban Development (HUD) and the Federal Office of Rural Health Policy (FORHP) [21, 24, 25]. For zip codes overlapping multiple counties, COVID-19 rates were weighted by the proportion of zip code residences in each associated county using crosswalk data files from HUD. Responses regarding test results for active COVD-19 infection and receipt of COVID-19 vaccine and booster shots were tabulated cumulatively from baseline and repeated surveys. Cumulative responses regarding test results for active COVD-19 infection were dichotomized as “Prior COVID-19 positive” or “No prior COVID-19 positive”. Cumulative responses regarding receipt of COVID-19 vaccine and booster shots were categorized as “None”, “1 or 2 doses” (primary vaccination), “vaccine + booster” (primary vaccination plus booster), or “No response” for participants who completed no prior survey questions about vaccination.

### Statistical analysis

All analyses were performed using R Statistical Software v4.3.2 or Stata v17. Counts of participants were tabulated according to baseline demographics, medical conditions, education and employment field. Participant frequencies stratified by subjective social status were compared using the chi-squared test. Monthly anxiety surveys completed and GAD-7 scores were then summarized according to baseline and time-varying characteristics. GAD-7 scores within covariate groups were compared using Welch’s ANOVA test. Frequencies for GAD-7 scores ≥ 10 within covariate groups were compared using the chi-squared test, or using Fisher’s exact test for unbalanced covariate groups with ≤ 100 observations in any subgroup. Plots of unadjusted anxiety scores over time were generated using the function “geom_smooth” from the R package ggplot2, with default settings for the smoothing method (mgcv::gam), spline basis (bs =“cs”; for cubic regression with shrinkage) and spline dimension (k = 10).

To analyze associations between each of our primary predictor variables and anxiety, we constructed a series of generalized additive mixed models (using the R packages “gamm4” v0.2–6, “mgcv” v1.9–1, and “lme4” v1.1–35.1 [26]) with the GAD-7 anxiety score as the outcome variable. For each primary predictor, we started with a simple model including only the predictor and calendar time, modeled as a cubic spline of days elapsed following declaration of COVID-19 as a global pandemic by the WHO on March 11, 2020, specified using gamm4 defaults with thin plate regression as the spline smoothing basis and k = 10 as the basis dimension. We then accounted for repeated measures from individual participants and differing individual tendencies towards anxiety by including a random intercept for each participant. Models were further adjusted by adding 1) baseline demographic variables, including medical conditions and living with school-aged children; 2) county-level COVID-19 case and death rates; and 3) personal COVID-19 experience including prior COVID-19 test positivity, vaccination status, monthly hospitalization days and emergency room/urgent care visits (without hospital admission). Quadratic terms for age and subjective social status were included to model non-linear relationships. To estimate their independent contributions, the fully adjusted models included both COVID-19 health worry and economic stress (difficulty paying for basic living expenses in primary analysis, or difficulty making ends meet in a supplemental analysis). To test for interactions between COVID-19 worry, economic difficulty and subjective social status, we added a three-way interaction term to the fully adjusted model with difficulty paying for basic living expenses as the primary predictor, dichotomizing subjective social status values below versus at or above the median in the interaction term. Estimates for the total combined effects of these variables and their interactions were obtained using the R package “multcomp”.

## Results

Between April 22, 2020 and May 4, 2022, 56,299 participants submitted a total of 371,076 anxiety surveys. After removing incomplete GAD-7 questionnaires (n = 188), participants reporting residence outside the United States (n = 1,634) and responses where key covariates were missing (n = 26,108), our analytic sample included 50,172 participants and 333,292 anxiety survey responses. These participants were predominantly non-Hispanic white (82%) and female (67%), with a median age of 55 (IQR 42–66). The majority of participants completed more than one anxiety survey (82%), accounting for 97% of the anxiety surveys analyzed (324,106). Approximately half of all participants were from Western census region states, with many from California (36%). A large proportion were highly educated with either a college degree (41%) or graduate degree (44%), and many reported employment in healthcare (18%) or education (12%). Participants reporting subjective social status at or above the median (≥7 on scale of 1–10) were more likely to report older age, male gender, higher levels of education, and non-rural residence (p < .0001 for all) (Table 1). Participants reporting lower subjective social status were more likely to report high levels of COVID-19 health worry (“Very worried” or “Extremely worried”; RR 1.24; 95% CI 1.21, 1.27; p < 0.0001) and much more likely to report high levels of difficulty paying for basic living expenses (“Hard” or “Very hard”; RR 5.11; 95% CI 4.73, 5.52; p < 0.0001) at least once during follow-up (Table 1 and S2 and S3 Tables).

**Table 1:** Baseline characteristics of participants stratified by subjective social status.

Characteristic	Value	Subjective Social Status 1–6 (n = 17518)	Subjective Social Status 7–10 (n = 32654)
Age group	18–34	3153 (18%)	3477 (10.6%)
	35–49	5786 (33%)	9757 (29.9%)
	50–64	5048 (28.8%)	10350 (31.7%)
	≥65	3531 (20.2%)	9070 (27.8%)
Gender	Female	12688 (72.4%)	20960 (64.2%)
	Male	4416 (25.2%)	11409 (34.9%)
	Genderqueer	157 (0.9%)	116 (0.4%)
	Another Gender Identity	111 (0.6%)	71 (0.2%)
	Not stated or decline to state	49 (0.3%)	48 (0.1%)
	Transgender Man	56 (0.3%)	33 (0.1%)
	Transgender Woman	41 (0.2%)	17 (0.1%)
Race	White	14630 (83.5%)	28835 (88.3%)
	Asian (including South Asian and Asian Indian)	736 (4.2%)	1655 (5.1%)
	More than one race	896 (5.1%)	1027 (3.1%)
	Black or African American	515 (2.9%)	421 (1.3%)
	Some other race	464 (2.6%)	380 (1.2%)
	Not stated	167 (1%)	249 (0.8%)
	American Indian or Alaska Native	45 (0.3%)	39 (0.1%)
	Don’t know	40 (0.2%)	24 (0.1%)
	Native Hawaiian or Pacific Islander	25 (0.1%)	24 (0.1%)
Hispanic ethnicity	No	15638 (89.3%)	30751 (94.2%)
	Yes: Mexican, Mexican American or Chicano	803 (4.6%)	716 (2.2%)
	Yes: Other or Mixed Hispanic, Latino or Spanish origin	666 (3.8%)	802 (2.5%)
	Yes: Puerto Rican	177 (1%)	139 (0.4%)
	Don’t know	114 (0.7%)	93 (0.3%)
	Prefer not to state	67 (0.4%)	87 (0.3%)
	Yes: Cuban	52 (0.3%)	65 (0.2%)
	Not stated	1 (0%)	1 (0%)
Education	No high school degree	76 (0.4%)	19 (0.1%)
	High school degree or equivalent	4253 (24.3%)	2541 (7.8%)
	College degree (including associate’s)	8190 (46.8%)	12511 (38.3%)
	Graduate degree	4742 (27.1%)	17356 (53.2%)
	Other	225 (1.3%)	182 (0.6%)
	Don’t know or not stated	32 (0.2%)	45 (0.1%)
Employment	Other or not stated	7273 (41.5%)	13953 (42.7%)
	Healthcare	3298 (18.8%)	5872 (18%)
	Education	2276 (13%)	3989 (12.2%)
	Scientific and technical services	1152 (6.6%)	3861 (11.8%)
	Finance and insurance	771 (4.4%)	1815 (5.6%)
	Arts, entertainment, and recreation	538 (3.1%)	813 (2.5%)
	Retail	624 (3.6%)	417 (1.3%)
	Manufacturing	398 (2.3%)	641 (2%)
	Hospitality and food services	437 (2.5%)	335 (1%)
	Construction	297 (1.7%)	389 (1.2%)
	Transportation	302 (1.7%)	344 (1.1%)
	Utilities	152 (0.9%)	225 (0.7%)
Children	Live with children (k-12 age)	4187 (23.9%)	7896 (24.2%)
Rural residence	Rural zip code	1936 (11.1%)	2346 (7.2%)
US Census Region	West	7856 (44.8%)	17027 (52.1%)
	Midwest	3796 (21.7%)	5419 (16.6%)
	Northeast	1850 (10.6%)	3490 (10.7%)
	South	4014 (22.9%)	6717 (20.6%)
	Territory	2 (0%)	1 (0%)
Baseline medical conditions	High blood pressure	5036 (28.7%)	8695 (26.6%)
	Diabetes	1454 (8.3%)	1757 (5.4%)
	Coronary artery disease	694 (4%)	1347 (4.1%)
	Heart attack	305 (1.7%)	513 (1.6%)
	Congestive heart failure	240 (1.4%)	284 (0.9%)
	Stroke	407 (2.3%)	590 (1.8%)
	Atrial fibrillation	647 (3.7%)	1409 (4.3%)
	Sleep apnea	2604 (14.9%)	3974 (12.2%)
	COPD	597 (3.4%)	558 (1.7%)
	Asthma	2161 (12.3%)	2748 (8.4%)
	Cancer	974 (5.6%)	2178 (6.7%)
	Immunodeficiency	710 (4.1%)	857 (2.6%)
	HIV	151 (0.9%)	140 (0.4%)
	Anemia	2417 (13.8%)	3074 (9.4%)
	Pregnant at baseline	159 (0.9%)	350 (1.1%)
COVID-19 health worry	High levels of COVID-19 health worry^1^ reported at any time during follow-up	7648 (43.7%)	11479 (35.2%)
Difficulty paying for basic living expenses	High levels of difficulty paying for basics^2^ reported at any time during follow-up	2299 (13.1%)	839 (2.6%)

^1^ –COVID-19 health worry reported as “Very worried” or “Extremely worried”.

^2^ –Difficulty paying for basic living expenses reported as “Hard” or “Very hard”

The mean GAD-7 anxiety score was 4.0 +/- 4.6 overall, with 11.4% of all responses meeting criteria considered high risk for clinically significant generalized anxiety (GAD-7 score ≥ 10). Anxiety scores were generally higher among participants reporting lower subjective social status, younger age, non-male gender, non-White race (except for Black and Asian), Hispanic ethnicity, or lower education. Anxiety scores were also higher among participants living with children and with some types of medical conditions (respiratory conditions, immunodeficiency, anemia) (p < 0.001 for all comparisons, Table 2).

**Table 2:** Anxiety scores by baseline and time-varying characteristics.

Characteristic		Participants	Monthly surveys completed		GAD-7 anxiety score			
					Mean +/- SD		Proportion ≥ 10, %	
			Per participant, median (IQR)	Total		p-value^1^		p-value^2^
**Overall**		50172	5 (2, 9)	333292	4.0 +/- 4.6	..	11.4%	..
Subjective social status	1	117	3 (1, 6)	529	8.3 +/-7.2	< 0.0001	38.8%	< 0.0001
	2	283	4 (2, 8)	1637	7.8 +/-6.6		37.1%	
	3	1114	4 (1, 8)	6492	6.7 +/-5.8		27.2%	
	4	2577	4 (2, 8)	15488	5.8 +/-5.5		21.6%	
	5	5099	4 (2, 8)	30399	4.9 +/-5.1		16.0%	
	6	8328	5 (2, 9)	54476	4.4 +/-4.7		13.4%	
	7	13905	5 (2, 9)	93081	3.9 +/-4.4		10.5%	
	8	11873	5 (2, 10)	82779	3.4 +/-4.1		8.4%	
	9	5128	5 (2, 10)	36812	3.0 +/-3.8		6.9%	
	10	1748	5 (2, 10)	11599	2.6 +/-3.7		5.7%	
Age group	18–34	6630	3 (1, 7)	31738	6.2 +/-5.2	< 0.0001	22.4%	< 0.0001
	35–49	15543	4 (2, 9)	95606	5.2 +/-4.9		16.9%	
	50–64	15398	5 (2, 10)	111429	3.7 +/-4.4		9.6%	
	≥65	12601	6 (3, 10)	94519	2.4 +/-3.4		4.3%	
Gender	Female	33648	5 (2, 9)	224075	4.4 +/-4.7	< 0.0001	12.8%	< 0.0001
	Male	15825	5 (2, 9)	104868	3.0 +/-4.1		7.5%	
	Genderqueer	273	4 (2, 9)	1743	8.1 +/-5.6		33.8%	
	Another Gender Identity	182	5 (2, 9)	1136	8.3 +/-5.8		38.2%	
	Not stated or decline to state	97	3 (2, 7)	467	5.5 +/-4.8		20.6%	
	Transgender Man	89	5 (2, 11)	641	8.2 +/-5.1		35.6%	
	Transgender Woman	58	4 (2, 9)	362	5.8 +/-5.7		22.7%	
Race	White	43465	5 (2, 10)	294247	4.0 +/-4.5	< 0.0001	11.3%	< 0.0001
	Asian (including South Asian and Asian Indian)	2391	4 (2, 9)	14546	3.5 +/-4.3		9.0%	
	More than one race	1923	4 (2, 9)	11766	5.0 +/-5.2		16.9%	
	Black or African American	936	4 (2, 7)	4890	3.6 +/-4.5		10.9%	
	Some other race	844	3 (2, 7)	4344	4.7 +/-5.3		16.1%	
	Not stated	416	4 (2, 8)	2487	4.1 +/-4.5		11.4%	
	American Indian or Alaska Native	84	3 (2, 7)	420	4.8 +/-5.9		17.9%	
	Don’t know	64	4 (2, 7)	311	4.7 +/-5.6		18.0%	
	Native Hawaiian or Pacific Islander	49	4 (2, 8)	281	4.0 +/-4.7		11.0%	
Hispanic ethnicity	No	46389	5 (2, 9)	312117	3.9 +/-4.5	< 0.0001	11.1%	< 0.0001
	Yes: Mexican, Mexican American or Chicano	1519	4 (2, 9)	8767	5.0 +/-5.2		16.8%	
	Yes: Other or Mixed Hispanic, Latino or Spanish origin	1468	4 (2, 8)	8119	4.9 +/-5.3		16.5%	
	Yes: Puerto Rican	316	4 (2, 8)	1767	5.1 +/-5.4		17.0%	
	Don’t know	207	4 (2, 8)	1204	4.8 +/-4.9		16.4%	
	Prefer not to state	154	3.5 (1, 6)	755	3.6 +/-4.5		10.1%	
	Yes: Cuban	117	3 (1, 6)	551	5.0 +/-5.3		18.3%	
	Not stated	2	6 (4.5, 7.5)	12	2.1 +/-3.6		0.0%	
Education	No high school degree	95	3 (1.5, 7)	460	5.0 +/-5.1	< 0.0001	15.7%	< 0.0001
	High school degree or equivalent	6794	4 (2, 8)	39792	4.5 +/-5.2		15.1%	
	College degree (including associate’s)	20701	5 (2, 9)	134069	4.1 +/-4.6		11.6%	
	Graduate degree	22098	5 (2, 10)	155988	3.8 +/-4.4		10.2%	
	Other	407	5 (2, 9)	2538	4.4 +/-5.2		14.4%	
	Don’t know or not stated	77	5 (2, 8)	445	5.2 +/-5.6		20.0%	
Employment	Other or not stated	21226	5 (2, 10)	146529	3.6 +/-4.4	< 0.0001	9.8%	< 0.0001
	Healthcare	9170	4 (2, 9)	57745	4.2 +/-4.6		12.2%	
	Education	6265	5 (2, 10)	42999	4.7 +/-4.7		13.9%	
	Scientific and technical services	5013	5 (2, 10)	34509	4.1 +/-4.5		11.9%	
	Finance and insurance	2586	5 (2, 9)	16637	3.8 +/-4.4		10.4%	
	Arts, entertainment, and recreation	1351	5 (2, 10)	8780	5.1 +/-5.2		16.7%	
	Retail	1041	4 (1, 8)	5791	5.0 +/-5.2		17.1%	
	Manufacturing	1039	4 (2, 9)	6304	3.3 +/-4.1		8.1%	
	Hospitality and food services	772	4 (1, 8)	4407	5.0 +/-5.1		16.8%	
	Construction	686	4 (2, 8)	3835	4.0 +/-4.7		11.6%	
	Transportation	646	4 (2, 8)	3569	3.8 +/-4.9		11.9%	
	Utilities	377	4 (2, 8)	2187	4.2 +/-4.5		11.9%	
Children in household	Live with children	12083	4 (2, 8)	71725	4.7 +/-4.8	< 0.0001	14.3%	< 0.0001
	Do not live with children or other	38089	5 (2, 10)	261567	3.8 +/-4.5		10.6%	
Zip code	Rural zip code	4282	5 (2, 9)	28197	3.9 +/-4.6	0.0031	11.4%	0.8
	Non-rural zip code	45890	5 (2, 9)	305095	4.0 +/-4.6		11.4%	
US Census region	West	24883	5 (2, 10)	175739	4.0 +/-4.5	< 0.0001	11.1%	< 0.0001
	Midwest	9215	5 (2, 10)	60545	3.9 +/-4.5		11.2%	
	Northeast	5340	4 (2, 8)	33814	4.4 +/-4.8		13.1%	
	South	10731	4 (2, 7)	63182	4.0 +/-4.6		11.5%	
	Territory	3	3 (2, 5.5)	12	8.2 +/-4.3		16.7%	
High blood pressure	High blood pressure	13731	5 (2, 9)	94237	3.4 +/-4.4	< 0.0001	9.2%	< 0.0001
	No, don’t know or not stated	36441	5 (2, 9)	239055	4.2 +/-4.6		12.3%	
Diabetes	Diabetes	3211	5 (2, 8)	20553	3.7 +/-4.6	< 0.0001	10.6%	< 0.0001
	No, don’t know or not stated	46961	5 (2, 9)	312739	4.0 +/-4.6		11.5%	
Coronary artery disease	Coronary artery disease	2041	5 (3, 9)	13658	2.9 +/-4.2	< 0.0001	7.5%	< 0.0001
	No, don’t know or not stated	48131	5 (2, 9)	319634	4.0 +/-4.6		11.6%	
Heart attack	Heart attack	818	5 (3, 9)	5484	2.9 +/-4.2	< 0.0001	7.7%	< 0.0001
	No, don’t know or not stated	49354	5 (2, 9)	327808	4.0 +/-4.6		11.5%	
Congestive heart failure	Congestive heart failure	524	5 (2, 9)	3418	3.8 +/-4.8	0.067	11.7%	0.59
	No, don’t know or not stated	49648	5 (2, 9)	329874	4.0 +/-4.6		11.4%	
Stroke	Stroke	997	5 (2, 9)	6595	3.8 +/-4.6	0.00012	11.3%	0.66
	No, don’t know or not stated	49175	5 (2, 9)	326697	4.0 +/-4.6		11.4%	
Atrial fibrillation	Atrial fibrillation	2056	6 (3, 10)	15124	2.8 +/-3.9	< 0.0001	6.6%	< 0.0001
	No, don’t know or not stated	48116	5 (2, 9)	318168	4.1 +/-4.6		11.7%	
Sleep apnea	Sleep apnea	6578	5 (2, 9)	44303	4.0 +/-4.6	0.59	11.8%	0.0046
	No, don’t know or not stated	43594	5 (2, 9)	288989	4.0 +/-4.6		11.4%	
COPD	COPD	1155	5 (2, 9)	7391	4.4 +/-4.9	< 0.0001	13.4%	< 0.0001
	No, don’t know or not stated	49017	5 (2, 9)	325901	4.0 +/-4.6		11.4%	
Asthma	Asthma	4909	5 (2, 9)	31952	5.1 +/-5.1	< 0.0001	17.2%	< 0.0001
	No, don’t know or not stated	45263	5 (2, 9)	301340	3.9 +/-4.5		10.8%	
Cancer	Cancer	3152	6 (2, 10)	21734	3.1 +/-4.1	< 0.0001	7.0%	< 0.0001
	No, don’t know or not stated	47020	5 (2, 9)	311558	4.1 +/-4.6		11.7%	
Immuno-deficiency	Immunodeficiency	1567	5 (2, 8)	9538	4.8 +/-5.1	< 0.0001	16.6%	< 0.0001
	No, don’t know or not stated	48605	5 (2, 9)	323754	4.0 +/-4.6		11.3%	
HIV	HIV	291	5 (2, 10)	1933	4.2 +/-4.6	0.031	12.7%	0.091
	No, don’t know or not stated	49881	5 (2, 9)	331359	4.0 +/-4.6		11.4%	
Anemia	Anemia	5491	5 (2, 10)	37013	5.0 +/-5.0	< 0.0001	16.0%	< 0.0001
	No, don’t know or not stated	44681	5 (2, 9)	296279	3.9 +/-4.5		10.9%	
Pregnant at baseline	Pregnant at baseline	509	4 (2, 8)	2862	4.6 +/-4.4	< 0.0001	12.4%	0.093
	No, don’t know or not stated	49663	5 (2, 9)	330430	4.0 +/-4.6		11.4%	
Date interval	2020-04-22–2020-10-24	..	..	57964	5.1 +/-4.9	< 0.0001	16.2%	< 0.0001
	2020-10-25–2021-04-28	..	..	72270	4.6 +/-4.8		14.0%	
	2021-04-29–2021-10-30	..	..	93567	3.6 +/-4.3		9.4%	
	2021-10-31–2022-05-04	..	..	109491	3.4 +/-4.3		8.9%	
COVID-19 cases per 100k	< 7	..	..	81119	4.0 +/-4.5	< 0.0001	11.2%	< 0.0001
	7–15	..	..	83918	3.9 +/-4.5		11.1%	
	> 15–34	..	..	84806	4.1 +/-4.6		11.7%	
	> 34	..	..	83449	4.0 +/-4.6		11.6%	
COVID-19 deaths per 100k	< 0.07	..	..	72992	3.8 +/-4.5	< 0.0001	10.6%	< 0.0001
	0.7–0.17	..	..	95007	4.0 +/-4.5		11.1%	
	> 0.17–0.36	..	..	82438	4.0 +/-4.6		11.5%	
	> 0.36	..	..	82855	4.2 +/-4.7		12.4%	
Difficulty paying for basic living expenses	Not very hard	..	..	298138	3.7 +/-4.3	< 0.0001	9.6%	< 0.0001
	Somewhat hard	..	..	22580	6.5 +/-5.5		24.9%	
	Hard	..	..	4859	8.3 +/-6.0		36.6%	
	Very hard	..	..	3220	9.8 +/-6.7		47.0%	
	Prefer not to state	..	..	3046	4.0 +/-4.9		12.2%	
	Don’t know	..	..	1449	5.9 +/-5.7		23.1%	
Difficulty making ends meet	Never	..	..	266181	3.5 +/-4.2	< 0.0001	8.7%	< 0.0001
	Hardly ever	..	..	35337	5.1 +/-5.0		16.8%	
	Occasionally	..	..	20837	6.7 +/-5.6		25.8%	
	Frequently	..	..	6295	9.2 +/-6.4		43.1%	
	Prefer not to state	..	..	3056	4.5 +/-5.2		15.0%	
	Don’t know	..	..	1586	6.2 +/-5.6		24.2%	
Daily activities	Working full time	..	..	170824	4.4 +/-4.6	< 0.0001	12.9%	< 0.0001
	Working part-time	..	..	35153	3.9 +/-4.5		10.6%	
	Unemployed, laid off, or looking for work	..	..	10968	6.2 +/-5.6		23.5%	
	In school (full- or part-time student)	..	..	5770	6.3 +/-5.4		24.4%	
	Stay-at-home parent or keeping household	..	..	18825	5.0 +/-5.0		15.9%	
	Retired	..	..	84259	2.3 +/-3.3		4.0%	
	Disabled	..	..	6180	6.7 +/-5.8		27.6%	
	Prefer not to state	..	..	1313	4.8 +/-5.4		17.1%	
Income change	Yes, it has increased	..	..	19073	4.2 +/-4.6	< 0.0001	12.4%	< 0.0001
	No, it is about the same	..	..	289182	3.8 +/-4.4		10.5%	
	Yes, it has declined	..	..	22229	6.2 +/-5.6		22.7%	
	Prefer not to state	..	..	2808	4.5 +/-5.3		15.5%	
Working from home	None	..	..	52452	4.3 +/-4.8	< 0.0001	13.2%	< 0.0001
	1–24% of the time	..	..	20492	4.1 +/-4.5		11.2%	
	25–49% of the time	..	..	8937	4.2 +/-4.4		11.8%	
	50–74% of the time	..	..	13066	4.2 +/-4.4		11.6%	
	75–99% of the time	..	..	24666	4.3 +/-4.5		12.1%	
	100% of the time	..	..	86355	4.4 +/-4.6		12.8%	
	Not stated or not applicable	..	..	127324	3.4 +/-4.5		9.6%	
COVID-19 test positivity	No prior COVID-19 positive	..	..	312458	4.0 +/-4.6	0.0029	11.4%	0.37
	Prior COVID-19 positive	..	..	20834	3.9 +/-4.7		11.6%	
COVID-19 vaccination	None	..	..	112081	4.6 +/-4.8	< 0.0001	13.9%	< 0.0001
	1 or 2 doses	..	..	96175	3.6 +/-4.3		9.5%	
	Vaccine + booster	..	..	99446	3.3 +/-4.2		8.6%	
	No response	..	..	25590	5.6 +/-5.2		18.9%	
Hospitalization days or ER/urgent care visits in previous month	None or not stated	..	..	321447	4.0 +/-4.5	< 0.0001	11.2%	< 0.0001
	One	..	..	8823	4.9 +/-5.0		16.0%	
	More than one	..	..	3022	5.2 +/-5.3		17.7%	
Worry about the health effects of COVID-19	Not worried at all	..	..	49720	1.8 +/-3.2	< 0.0001	3.5%	< 0.0001
	A little worried	..	..	110795	2.7 +/-3.5		4.8%	
	Somewhat worried	..	..	104670	4.3 +/-4.2		11.0%	
	Very worried	..	..	48514	6.4 +/-5.0		22.5%	
	Extremely worried	..	..	19593	9.4 +/-6.2		43.7%	

IQR–Interquartile range; SD–Standard deviation; GAD-7 anxiety score–Anxiety score (scale 0–21) from the Generalized Anxiety Disorder (GAD-7) questionnaire

^1^ Welch’s ANOVA for homogeneity of mean GAD-7 scores

^2^ chi-squared test or Fisher’s exact test for frequencies of GAD-7 scores ≥ 10; Fisher’s exact test was used for unbalanced groups with ≤ 100 observations for any subgroup

**Fig 1:**
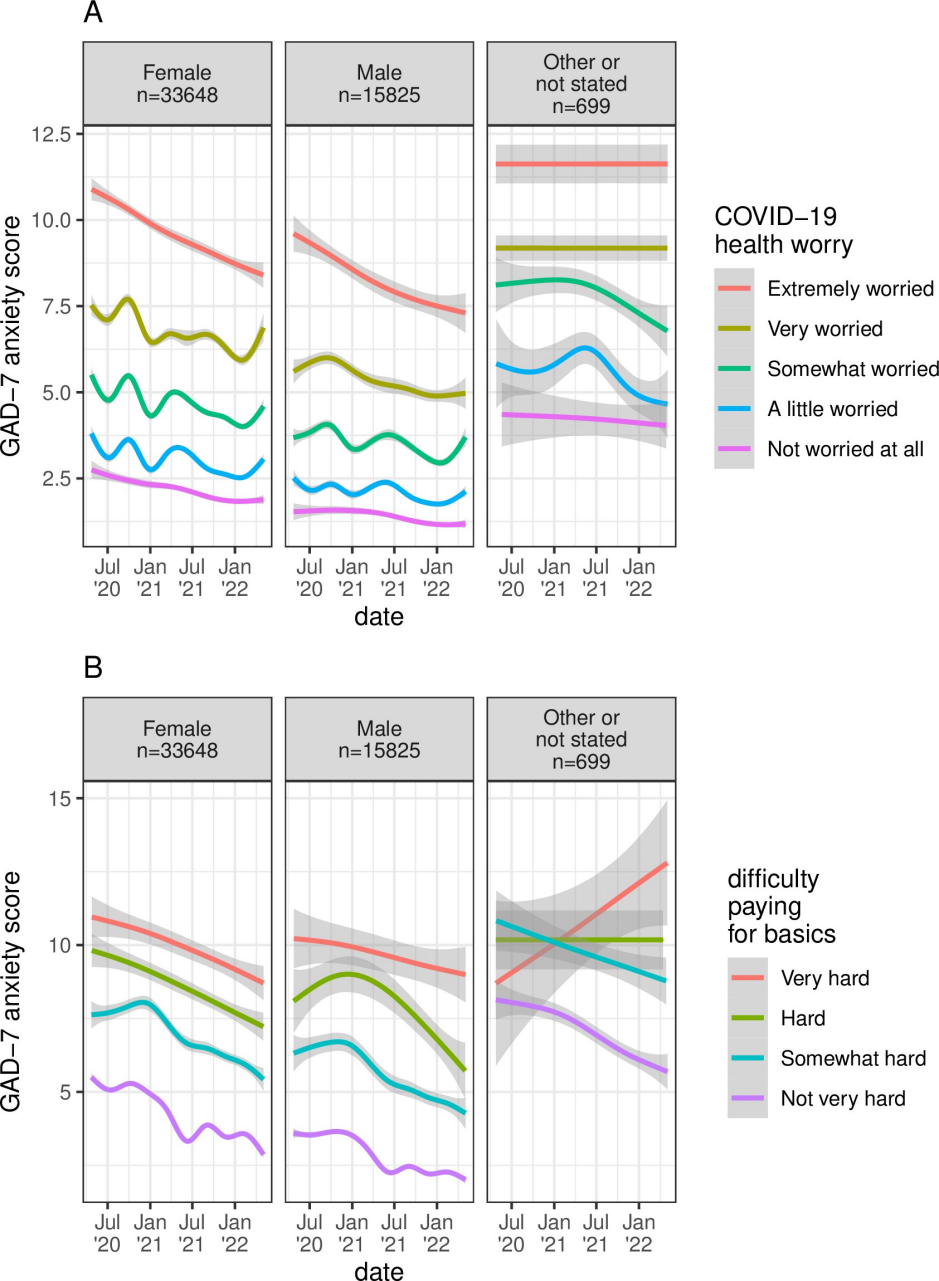
GAD-7 anxiety scores over the first two years of the pandemic. Anxiety scores divided by gender are further stratified by worry about the health effects of COVID-19 in panel A, and by difficulty paying for basic living expenses in panel B. Trendlines show unadjusted mean GAD-7 anxiety scores, with 95% confidence intervals shaded in grey. Counts in both panels show total observations in each gender group, including observations associated with non-informative levels of difficulty paying for basic living expenses (“Prefer not to state”, “Don’t know”), which are omitted in panel B.

**Fig 2:**
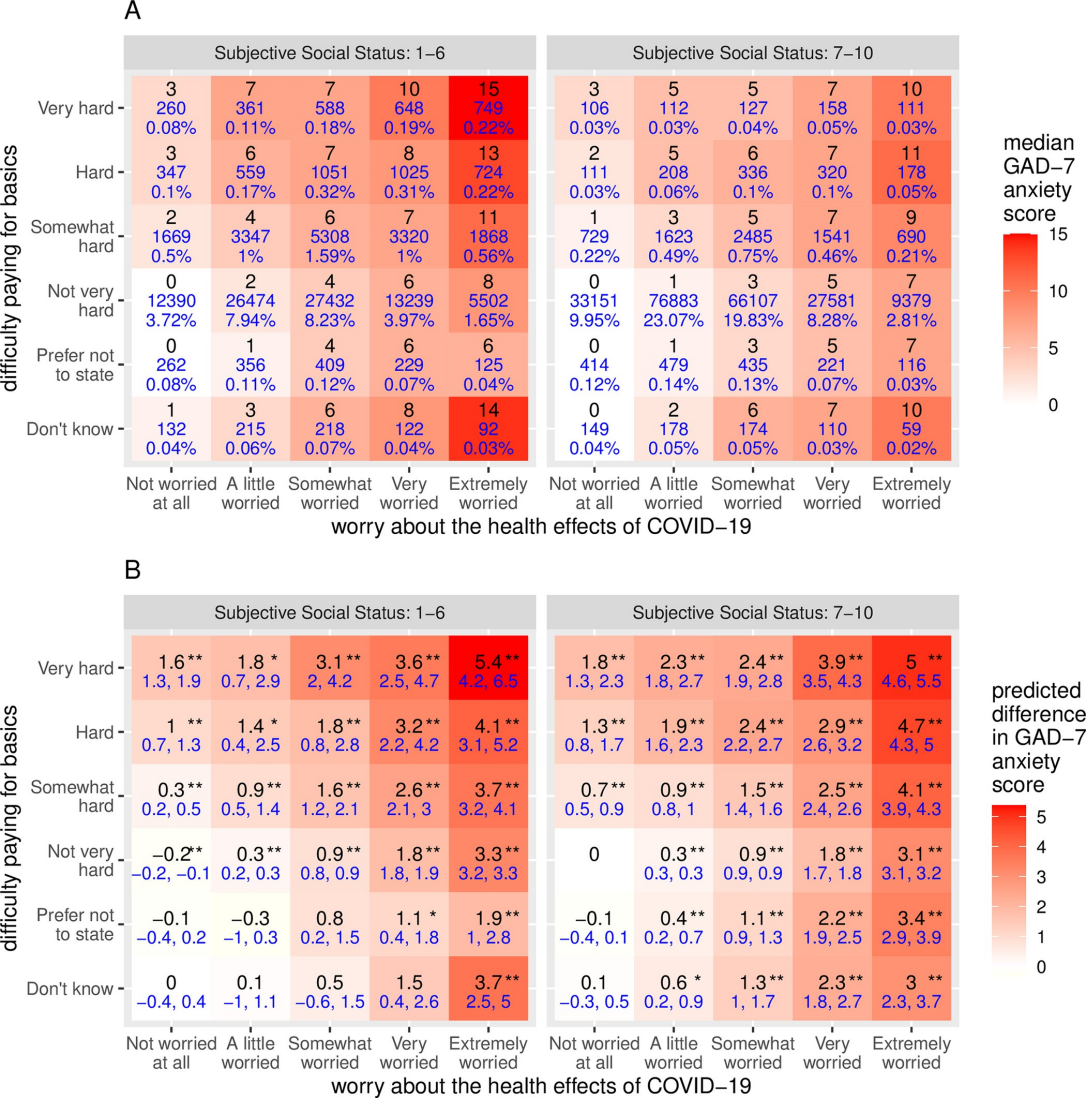
GAD-7 anxiety scores and associations with COVID-19 health worry, economic stress and subjective social status. Panel A shows median GAD-7 scores (black) and total observation counts and percentages (blue) cross-tabulated by corresponding responses regarding COVID-19 health worry and difficulty paying for basic living expenses, and stratified by lower versus higher subjective social status. Panel B shows model estimates from a multivariable regression model that includes interactions between these variables. Estimates (black) and 95% confidence intervals (blue) indicate model predictions relative to reference levels of COVID-19 health worry (“Not very worried”), difficulty paying for basics (“Not very hard”) and subjective social status (values between 7–10). * p < 0.01, ** p < 0.001.

Anxiety was variable across the pandemic and within participants over time. The mean range in GAD-7 anxiety scores was 4.9 +/- 4.2 (SD) points among participants who completed more than one anxiety survey. Responses earlier in the pandemic were associated with higher anxiety scores (Table 2), with subsequent fluctuations corresponding to successive waves of the pandemic and their repercussions in the US (Fig 1). Prior positive tests for active COVID-19 infection and county-level COVID-19 case and death rates were not associated with substantially elevated anxiety scores, but receipt of COVID-19 vaccinations was associated with lower anxiety scores. Worry about the health effects of COVID-19 for oneself or loved ones was associated with highly elevated anxiety scores (Table 2). Time-varying economic factors were also very strongly associated with elevated anxiety scores. These include decline in income, disability, unemployment, full or part-time student status, and difficulty paying for basic living expenses or making ends meet. More than 40% of responses among participants reporting the highest levels of COVID-19 health worry or economic difficulty were associated with GAD-7 scores indicating high risk for clinically significant generalized anxiety (GAD-7 ≥ 10) (Table 2). Participants reporting high levels of *both* COVID-19 health worry and difficulty paying for basics were particularly prone to anxiety, with median scores considered high risk for generalized anxiety (GAD-7 ≥ 10) in many of the highest categories of exposure, and median scores indicative of severe anxiety (GAD-7 ≥ 15) at the highest levels of exposure among those also reporting lower subjective social status (Fig 2).

COVID-19 health worry and difficulty paying for basic living expenses were both independently associated with elevated GAD-7 anxiety scores in multivariable regression models (Table 3). After accounting for the general tendency for each participant to report higher or lower GAD-7 scores (Model 2), and adjusting for other covariates (Model 3), highly significant dose-response trends were evident (p < 2e-16 for each), adding 2–3 additional points to GAD-7 scores in highest categories of exposure (Table 3 and S4 Table). Lower subjective social status was also independently associated with higher GAD-7 scores in fully adjusted models (p < 2e-16). To investigate potential synergistic effects between economic stress, COVID-19 health worry and subjective social status we added interactions to the fully adjusted model shown in Table 3, dichotomizing subjective social status values above or below the median in the interaction terms. The effects of COVID-19 health worry and difficulty paying for basics were independent and additive (p < 2e-16 for each) but not strongly synergistic in this model with interactions included, predicting GAD-7 scores 5.4 or 5.0 points higher at the highest levels of exposure for lower or higher subjective social status participants, respectively (Fig 2 and S5 Table).

**Table 3:** Associations between COVID-19 health worry, difficulty paying for basic living expenses, and anxiety during the COVID-19 pandemic.

COVID-19 Health Worry	GAD-7 Score difference (95% confidence intervals)^1^		
	Model 1: Adjusted for calendar time^2^	Model 2: Adjustment for participant tendencies^3^	Model 3: Fully adjusted^4^
Not worried or other	0 (reference)	0 (reference)	0 (reference)
A little worried	0.88 (0.84, 0.93)	0.38 (0.35, 0.40)	0.36 (0.33, 0.39)
Somewhat worried	2.49 (2.45, 2.53)	1.00 (0.97, 1.03)	0.95 (0.92, 0.98)
Very worried	4.57 (4.52, 4.62)	1.95 (1.92, 1.98)	1.88 (1.84, 1.91)
Extremely worried	7.51 (7.44, 7.58)	3.39 (3.34, 3.43)	3.28 (3.24, 3.33)
p-value for trend	p < 2e-16	p < 2e-16	p < 2e-16
adjusted R^2^	0.19	0.14	0.26
Difficulty Paying for Basics^5^			
Not very hard	0 (reference)	0 (reference)	0 (reference)
Somewhat hard	2.86 (2.80, 2.92)	0.86 (0.83, 0.90)	0.67 (0.63, 0.70)
Hard	4.63 (4.5, 4.75)	1.74 (1.67, 1.81)	1.36 (1.29, 1.43)
Very hard	6.11 (5.96, 6.26)	2.57 (2.48, 2.66)	2.06 (1.97, 2.15)
p-value for trend	p < 2e-16	p < 2e-16	p < 2e-16
adjusted R^2^	0.08	0.06	0.26

^1^ –Estimates and 95% confidence intervals represent the estimated difference in the Generalized Anxiety Disorder (GAD-7) score (scale 0–21) compared to the indicated reference category from multivariable linear regression models. p < 2e-16 for all point estimates shown.

^2^ –Days elapsed following declaration of COVID-19 as a global pandemic by the WHO on March 11, 2020.

^3^ –Adjusted for repeated measures from individual participants.

^4^ –Additionally adjusted for baseline demographic characteristics, medical conditions, subjective social status, time-varying COVID-19 case and death rates (associated with participant zip code), personal COVID-19 experience and vaccination status, and the other independent variable of interest (both Economic Stress and COVID-19 Worry were included in the model, unlike for Models 1 and 2). No interaction terms are included.

^5^ –“Prefer not to state” and “Don’t know” categories for economic stress are omitted from the table.

GAD-7—Generalized Anxiety Disorder (GAD-7) questionnaire

A version of the complete dataset used for all analyses is provided with minor modifications for anonymization (S1 File and S6 Table provides corresponding regression model results).

## Discussion

Our analysis of more than 330,000 survey responses from over 50,000 participants in the COVID-19 Citizen Science study shows high levels of anxiety early during the pandemic, with fluctuating declines over the subsequent two years. Worry about the health effects of COVID-19 was strongly associated with anxiety, predicting GAD-7 anxiety scores up to three points higher after adjustment for individual tendencies towards anxiety and other covariates. Economic difficulty was an independent contributor, predicting GAD-7 anxiety scores up to two points higher. The additive effects of COVID-19 health worry and economic difficulty were substantial; participants reporting both had very high levels of anxiety. While the effects of COVID-19 health worry and economic difficulty on anxiety were similar in magnitude for participants reporting lower or higher socioeconomic status, these factors were both disproportionately common in participants with subjectively lower social status.

Anxiety caused substantial suffering during the height of the pandemic. COVID-19 mortality in the US dropped from a peak of nearly 26,000 deaths per week in January 2021 to approximately 1,500 deaths per week as of January 2024. Most restrictive measures have been eased, and many support programs have expired. However, the impacts of the pandemic on physical, mental, and economic well-being remain profound. Our findings underscore previous work documenting the compounding and unequal effects of COVID-19 on physical health, employment, and financial insecurity among socioeconomically disadvantaged and marginalized groups, and the corresponding potential repercussions for mental health [4, 8, 10, 14, 15, 27]. Our analysis also highlights the vulnerability of people for whom health worry and financial difficulty are potentially in conflict, including those who do not have financial resources to find alternatives when their jobs or housing pose health risks, and those lacking access to affordable healthcare [9, 14, 28]. In our study, difficulty paying for basic living expenses and worry about the health effects of COVID-19 were associated with comparable increases in anxiety, and high levels of economic stress were much more common among socioeconomically vulnerable participants, suggesting that economic distress might outweigh health concerns for at least some people facing difficult decisions when these factors were in conflict.

Our analysis had limitations. We did not investigate all factors that might be important contributors to anxiety, such as social isolation or preexisting mental health conditions. Non-Hispanic white participants comprised the overwhelming majority in this study, limiting our ability to draw insights that might be distinct for other racial and ethnic groups. Roughly half of all participants were from Western states, and many of these were concentrated in metropolitan areas surrounding the San Francisco Bay area in California. In addition, less than 9% of participants in this study were from rural areas, and only 35% reported subjective social status values in the lower two-thirds of the MacArthur scale, underscoring the need for innovations in recruitment to reach more diverse participants for digitally based studies. Only 6% of CCS anxiety surveys were associated with a previous or concurrent positive test for SARS-CoV-2 infection, which may reflect low case rates in the cohort, low rates of testing [29], or both. We did not find evidence that the combined effects of economic difficulty, worry about the health effects of COVID-19, and subjective social status were strongly synergistic, but there are many other potential interactions involving participant characteristics and experiences that might merit investigation as important contributors to anxiety. Lastly, participation was likely influenced by important variables of interest, including anxiety, economic difficulty and worry about COVID-19. In each case, effects might be complex, for example increasing participation at moderate levels of distress while decreasing participation at the highest levels. Despite these limitations, the CCS study presents a unique resource for analyzing mental health over the course of the pandemic in the context of a wide range of participant characteristics and experiences, and our findings have important policy and public health implications.

## Conclusions

Our analyses indicate that mitigation of health risks and economic precarity for the most vulnerable could substantially reduce anxiety related to effects of the pandemic. Our findings also suggest that addressing only one of these factors without the other would leave many at risk for anxiety. Directly addressing rational concerns about health risk and economic insecurity could alleviate pandemic-related anxiety for many [7, 28]. Potential mitigating interventions range from measures to address workplace infection risk including guaranteed paid sick leave and improved indoor air quality, to supports for housing security, expanded access to affordable healthcare and mental health support services, community-centered vaccination campaigns, and programs to support basic levels of income [28, 30]. Such efforts could be instrumental for addressing continuing waves of COVID-19, and future public health emergencies, with potential co-benefits for addressing existing socioeconomic disparities in physical, mental, and economic health.

## Supporting information

S1 File Anonymized dataset. (ZIP)

S1 Table GAD-7 anxiety survey questionnaire and scoring. (PDF)

S2 Table Cross-tabulation of observations by participant subjective social status and time-varying worry about the health effects of COVID-19. (PDF)

S3 Table Cross-tabulation of observations by participant subjective social status and time-varying difficulty paying for basic living expenses. (PDF)

S4 Table Associations between COVID-19 health worry, difficulty making ends meet and anxiety during the COVID-19 pandemic. (PDF)

S5 Table Omnibus p–values from multivariable linear regression model with interactions between COVID-19 health worry, difficulty paying for basics, and subjective social status. (PDF)

S6 Table Associations between COVID-19 health worry, difficulty paying for basic living expenses, and anxiety during the COVID-19 pandemic using the anonymized S1 File. (PDF)

## References

[1] Collaborators C-MD. Global prevalence and burden of depressive and anxiety disorders in 204 countries and territories in 2020 due to the COVID-19 pandemic. Lancet. 2021;398(10312):1700–12. Epub 20211008. doi: 10.1016/S0140-6736(21)02143-7 ; PubMed Central PMCID: PMC8500697. 34634250 PMC8500697

[2] Kessler RC, Chiu WT, Hwang IH, Puac-Polanco V, Sampson NA, Ziobrowski HN, et al. Changes in Prevalence of Mental Illness Among US Adults During Compared with Before the COVID-19 Pandemic. Psychiatr Clin North Am. 2022;45(1):1–28. Epub 20211112. doi: 10.1016/j.psc.2021.11.013 ; PubMed Central PMCID: PMC8585610. 35219431 PMC8585610

[3] Household Pulse Survey—Anxiety and Depression October 25, 2022. Available from: https://www.cdc.gov/nchs/covid19/pulse/mental-health.htm.

[4] Panchal N, Kamal R, Cox C, Garfield R. The Implications of COVID-19 for Mental Health and Substance Use. October 26, 2021. Available from: https://www.kff.org/coronavirus-covid-19/issue-brief/the-implications-of-covid-19-for-mental-health-and-substance-use/.

[5] Taquet M, Geddes JR, Husain M, Luciano S, Harrison PJ. 6-month neurological and psychiatric outcomes in 236 379 survivors of COVID-19: a retrospective cohort study using electronic health records. Lancet Psychiatry. 2021;8(5):416–27. Epub 20210406. doi: 10.1016/S2215-0366(21)00084-5 ; PubMed Central PMCID: PMC8023694. 33836148 PMC8023694

[6] Xie Y, Xu E, Al-Aly Z. Risks of mental health outcomes in people with covid-19: cohort study. BMJ. 2022;376:e068993. Epub 20220216. doi: 10.1136/bmj-2021-068993 ; PubMed Central PMCID: PMC8847881. 35172971 PMC8847881

[7] Kampfen F, Kohler IV, Ciancio A, Bruine de Bruin W, Maurer J, Kohler HP. Predictors of mental health during the Covid-19 pandemic in the US: Role of economic concerns, health worries and social distancing. PLoS One. 2020;15(11):e0241895. Epub 20201111. doi: 10.1371/journal.pone.0241895 ; PubMed Central PMCID: PMC7657497. 33175894 PMC7657497

[8] Burns A. Will Long COVID Exacerbate Existing Disparities in Health and Employment? September 23, 2022. Available from: https://www.kff.org/policy-watch/will-long-covid-exacerbate-existing-disparities-in-health-and-employment/.

[9] Riley AR, Chen YH, Matthay EC, Glymour MM, Torres JM, Fernandez A, et al. Excess mortality among Latino people in California during the COVID-19 pandemic. SSM Popul Health. 2021;15:100860. Epub 20210702. doi: 10.1016/j.ssmph.2021.100860 ; PubMed Central PMCID: PMC8283318. 34307826 PMC8283318

[10] Kirzinger A, Sparks G, Hamel L, Stokes M, Montero A, Brodie M. KFF COVID-19 Vaccine Monitor: The Pandemic’s Toll on Workers and Family Finances During the Omicron Surge. March 10, 2022. Available from: https://www.kff.org/coronavirus-covid-19/poll-finding/kff-covid-19-vaccine-monitor-economic-impact/.

[11] Magesh S, John D, Li WT, Li Y, Mattingly-App A, Jain S, et al. Disparities in COVID-19 Outcomes by Race, Ethnicity, and Socioeconomic Status: A Systematic-Review and Meta-analysis. JAMA Netw Open. 2021;4(11):e2134147. Epub 20211101. doi: 10.1001/jamanetworkopen.2021.34147 ; PubMed Central PMCID: PMC8586903. 34762110 PMC8586903

[12] Aschmann HE, Riley AR, Chen R, Chen YH, Bibbins-Domingo K, Stokes AC, et al. Dynamics of racial disparities in all-cause mortality during the COVID-19 pandemic. Proc Natl Acad Sci U S A. 2022;119(40):e2210941119. Epub 20220920. doi: 10.1073/pnas.2210941119 ; PubMed Central PMCID: PMC9546535. 36126098 PMC9546535

[13] Risk for COVID-19 Infection, Hospitalization, and Death By Race/Ethnicity. December 8, 2022. Available from: https://www.cdc.gov/coronavirus/2019-ncov/covid-data/investigations-discovery/hospitalization-death-by-race-ethnicity.html.

[14] Goldman N, Pebley AR, Lee K, Andrasfay T, Pratt B. Racial and ethnic differentials in COVID-19-related job exposures by occupational standing in the US. PLoS One. 2021;16(9):e0256085. Epub 20210901. doi: 10.1371/journal.pone.0256085 ; PubMed Central PMCID: PMC8409606. 34469440 PMC8409606

[15] Goda GS, Soltas E. The Impacts of Covid-19 Illnesses on Workers. National Bureau of Economic Research Working Paper Series. 2022;No. 30435. doi: 10.3386/w30435

[16] Falk G, Romero P, Nicchitta I, Nyhof E. Unemployment Rates During the COVID-19 Pandemic. December 15, 2022. Available from: https://crsreports.congress.gov/product/pdf/R/R46554/21.

[17] Beatty AL, Peyser ND, Butcher XE, Carton TW, Olgin JE, Pletcher MJ, et al. The COVID-19 Citizen Science Study: Protocol for a Longitudinal Digital Health Cohort Study. JMIR Res Protoc. 2021;10(8):e28169. Epub 20210830. doi: 10.2196/28169 ; PubMed Central PMCID: PMC8407439. 34310336 PMC8407439

[18] Eureka Research Platform November 16, 2022. Available from: https://info.eurekaplatform.org/.

[19] Forrest CB, McTigue KM, Hernandez AF, Cohen LW, Cruz H, Haynes K, et al. PCORnet(R) 2020: current state, accomplishments, and future directions. J Clin Epidemiol. 2021;129:60–7. Epub 20200928. doi: 10.1016/j.jclinepi.2020.09.036 ; PubMed Central PMCID: PMC7521354. 33002635 PMC7521354

[20] Adler NE, Epel ES, Castellazzo G, Ickovics JR. Relationship of subjective and objective social status with psychological and physiological functioning: preliminary data in healthy white women. Health Psychol. 2000;19(6):586–92. doi: 10.1037//0278-6133.19.6.586 . 11129362

[21] COVID-19 Cases and Deaths Rolling Averages—County Level. The New York Times [Internet]. 2022 May 11, 2022. Available from: https://github.com/nytimes/covid-19-data/tree/master/rolling-averages.

[22] Lowe B, Decker O, Muller S, Brahler E, Schellberg D, Herzog W, et al. Validation and standardization of the Generalized Anxiety Disorder Screener (GAD-7) in the general population. Med Care. 2008;46(3):266–74. doi: 10.1097/MLR.0b013e318160d093 . 18388841

[23] Spitzer RL, Kroenke K, Williams JB, Lowe B. A brief measure for assessing generalized anxiety disorder: the GAD-7. Arch Intern Med. 2006;166(10):1092–7. Epub 2006/05/24. doi: 10.1001/archinte.166.10.1092 . 16717171

[24] HUD USPS ZIP CODE CROSSWALK FILES 2021 January 3, 2022; (3rd quarter 2021, September 20, 2021). Available from: https://www.huduser.gov/portal/datasets/usps_crosswalk.html.

[25] Federal Office of Rural Health Policy (FORHP) Data Files 2021 January 11, 2022. Available from: https://www.hrsa.gov/rural-health/about-us/definition/datafiles.html.

[26] Wood S, Scheipl F. gamm4: Generalized Additive Mixed Models using ’mgcv’ and ’lme4’2020 July 15, 2022. Available from: https://CRAN.R-project.org/package=gamm4.

[27] Hertz-Palmor N, Moore TM, Gothelf D, DiDomenico GE, Dekel I, Greenberg DM, et al. Association among income loss, financial strain and depressive symptoms during COVID-19: Evidence from two longitudinal studies. J Affect Disord. 2021;291:1–8. Epub 20210505. doi: 10.1016/j.jad.2021.04.054 ; PubMed Central PMCID: PMC8460400. 34022550 PMC8460400

[28] Rose N, Manning N, Bentall R, Bhui K, Burgess R, Carr S, et al. The social underpinnings of mental distress in the time of COVID-19—time for urgent action. Wellcome Open Res. 2020;5:166. Epub 20200713. doi: 10.12688/wellcomeopenres.16123.1 ; PubMed Central PMCID: PMC7411522. 32802967 PMC7411522

[29] Pletcher MJ, Olgin JE, Peyser ND, Modrow MF, Lin F, Martin J, et al. Factors Associated With Access to and Timing of Coronavirus Testing Among US Adults After Onset of Febrile Illness. JAMA Netw Open. 2021;4(5):e218500. Epub 20210503. doi: 10.1001/jamanetworkopen.2021.8500 ; PubMed Central PMCID: PMC8094007. 33938937 PMC8094007

[30] Batra A, Jackson K, Hamad R. Effects Of The 2021 Expanded Child Tax Credit On Adults’ Mental Health: A Quasi-Experimental Study. Health Aff (Millwood). 2023;42(1):74–82. doi: 10.1377/hlthaff.2022.00733 ; PubMed Central PMCID: PMC10089297. 36623218 PMC10089297

