# Supplementary material for: Factors associated with anxiety during the first two years of the COVID-19 pandemic in the United States: An analysis of the COVID-19 Citizen Science study
Source: PLoS One. 2024 Feb 6;19(2):e0297922. doi: 10.1371/journal.pone.0297922 (PMC10846720; doi:10.1371/journal.pone.0297922)
Supplement: S1 Table — (PDF) [file pone.0297922.s002.pdf]

S1 Table. GAD-7 anxiety survey questionnaire and scoring

Over the last two weeks, how often have you been bothered by the following problems?	Not at all	Several days	More than half the days	Nearly every day
1. Feeling nervous, anxious, or on edge	0	+ 1	+ 2	+ 3
2. Not being able to stop or control worrying	0	+ 1	+ 2	+ 3
3. Worrying too much about different things	0	+ 1	+ 2	+ 3
4. Trouble relaxing	0	+ 1	+ 2	+ 3
5. Being so restless that it is hard to sit still	0	+ 1	+ 2	+ 3
6. Becoming easily annoyed or irritable	0	+ 1	+ 2	+ 3
7. Feeling afraid, as if something awful might happen	0	+ 1	+ 2	+ 3
