# Supplementary material for: Factors associated with anxiety during the first two years of the COVID-19 pandemic in the United States: An analysis of the COVID-19 Citizen Science study
Source: PLoS One. 2024 Feb 6;19(2):e0297922. doi: 10.1371/journal.pone.0297922 (PMC10846720; doi:10.1371/journal.pone.0297922)
Supplement: S2 Table — (PDF) [file pone.0297922.s003.pdf]

S2 Table. Cross-tabulation of observations by participant subjective social status and time-varying worry about the health effects of COVID-19.

subjective social status	Worry about the health effects of COVID-19[1]				
	Not worried at all	A little worried	Somewhat worried	Very worried	Extremely worried
1-6 (n=109021)	15060 (13.8%)	31312 (28.7%)	35006 (32.1%)	18583 (17%)	9060 (8.3%)
7-10 (n=224271)	34660 (15.5%)	79483 (35.4%)	69664 (31.1%)	29931 (13.3%)	10533 (4.7%)

[1] – Percentages show the proportion of observations within each subjective social status group.
