# Supplementary material for: Factors associated with anxiety during the first two years of the COVID-19 pandemic in the United States: An analysis of the COVID-19 Citizen Science study
Source: PLoS One. 2024 Feb 6;19(2):e0297922. doi: 10.1371/journal.pone.0297922 (PMC10846720; doi:10.1371/journal.pone.0297922)
Supplement: S3 Table — (PDF) [file pone.0297922.s004.pdf]

S3 Table. Cross-tabulation of observations by participant subjective social status and time-varying difficulty paying for basic living expenses.

subjective social status	Difficulty paying for basic living expenses[1]					
	Not very hard	Somewhat hard	Hard	Very hard	Prefer not to state	Don't know
1-6 (n=109021)	85037 (79.6%)	15512 (14.5%)	3706 (3.5%)	2606 (2.4%)	1381 (1.3%)	779 (0.7%)
7-10 (n=224271)	213101 (96%)	7068 (3.2%)	1153 (0.5%)	614 (0.3%)	1665 (0.7%)	670 (0.3%)

[1] – Percentages show the proportion of observations within each subjective social status group.
