# Supplementary material for: Factors associated with anxiety during the first two years of the COVID-19 pandemic in the United States: An analysis of the COVID-19 Citizen Science study
Source: PLoS One. 2024 Feb 6;19(2):e0297922. doi: 10.1371/journal.pone.0297922 (PMC10846720; doi:10.1371/journal.pone.0297922)
Supplement: S4 Table — (PDF) [file pone.0297922.s005.pdf]

S4 Table. Associations between COVID-19 health worry, difficulty making ends meet, and anxiety during the COVID-19 pandemic.

	GAD-7 Score difference (95% confidence intervals)[1]		
**COVID-19 Health Worry**	**Model 1: Adjusted for calendar time[2]**	**Model 2: Adjustment for participant tendencies[3]**	**Model 3: Fully adjusted[4]**
Not worried or other	0 (reference)	0 (reference)	0 (reference)
A little worried	0.88 (0.84, 0.93)	0.38 (0.35, 0.40)	0.36 (0.33, 0.38)
Somewhat worried	2.49 (2.45, 2.53)	1.00 (0.97, 1.03)	0.95 (0.92, 0.97)
Very worried	4.57 (4.52, 4.62)	1.95 (1.92, 1.98)	1.88 (1.84, 1.91)
Extremely worried	7.51 (7.44, 7.58)	3.39 (3.34, 3.43)	3.28 (3.24, 3.33)
p-value for trend	p < 2e-16	p < 2e-16	p < 2e-16
adjusted R^2	0.19	0.14	0.26
**Difficulty Making Ends Meet[5]**			
Never	0 (reference)	0 (reference)	0 (reference)
Hardly ever	1.64 (1.60, 1.69)	0.46 (0.43, 0.49)	0.37 (0.34, 0.40)
Occasionally	3.20 (3.14, 3.26)	1.16 (1.13, 1.20)	0.92 (0.89, 0.96)
Frequently	5.70 (5.59, 5.81)	2.33 (2.26, 2.39)	1.88 (1.82,1.95)
p-value for trend	p < 2e-16	p < 2e-16	p < 2e-16
adjusted R^2	0.09	0.06	0.26

[1] – Estimates and 95% confidence intervals represent the estimated difference in the Generalized Anxiety Disorder (GAD-7) score (scale 0-21) compared to the indicated reference category from multivariable linear regression models. p < 2e-16 for all point estimates shown.

[2] – Days elapsed following declaration of COVID-19 as a global pandemic by the WHO on March 11, 2020.

[3] – Adjusted for repeated measures from individual participants.

[4] – Additionally adjusted for baseline demographic characteristics, medical conditions, subjective social status, time-varying COVID-19 case and death rates (associated with participant zip code), personal COVID-19 experience and vaccination status, and the other independent variable of interest (both Economic Stress and COVID-19 Worry were included in the model, unlike for Models 1 and 2). No interaction terms are included.

[5] – "Prefer not to state" and "Don't know" categories for economic stress are omitted from the table.

GAD-7 - Generalized Anxiety Disorder (GAD-7) questionnaire
