# Supplementary material for: Factors associated with anxiety during the first two years of the COVID-19 pandemic in the United States: An analysis of the COVID-19 Citizen Science study
Source: PLoS One. 2024 Feb 6;19(2):e0297922. doi: 10.1371/journal.pone.0297922 (PMC10846720; doi:10.1371/journal.pone.0297922)
Supplement: S5 Table — (PDF) [file pone.0297922.s006.pdf]

S5 Table. Omnibus p–values from multivariable linear regression model with interactions between COVID-19 health worry, difficulty paying for basics, and subjective social status.

Term	Term type	p-value[1]
Worry about the health effects of COVID-19	covariate	< 2e-16
Difficulty paying for basics	covariate	< 2e-16
Subjective Social Status	covariate	< 2e-16
Subjective Social Status^2 (quadratic)	covariate	< 2e-16
Subjective Social Status – dichotomized	covariate	3.5e-10
Difficulty paying for basics : COVID-19 health worry	two-way interaction	0.011
Difficulty paying for basics : Subjective Social Status – dichotomized	two-way interaction	0.55
COVID-19 health worry : Subjective Social Status – dichotomized	two-way interaction	3.3e-11
Difficulty paying for basics : COVID-19 health worry : Subjective Social Status – dichotomized	three-way interaction	0.0095
Gender	covariate	< 2e-16
Age	covariate	< 2e-16
Age^2 (quadratic)	covariate	< 2e-16
Race	covariate	< 2e-16
Hispanic ethnicity	covariate	< 2e-16
Education	covariate	< 2e-16
Employment	covariate	< 2e-16
Live with children	covariate	< 2e-16
Rural zip code	covariate	2.6e-10
US Census region	covariate	< 2e-16
High blood pressure	covariate	< 2e-16
Diabetes	covariate	0.014
Coronary artery disease	covariate	0.0058
Heart attack	covariate	0.10
Congestive heart failure	covariate	0.0067
Stroke	covariate	< 2e-16
Atrial fibrillation	covariate	0.64
Sleep apnea	covariate	< 2e-16
COPD	covariate	< 2e-16
Asthma	covariate	< 2e-16
Cancer	covariate	1.64e-05
Immunodeficiency	covariate	< 2e-16
HIV	covariate	< 2e-16
Anemia	covariate	< 2e-16
Pregnant at baseline	covariate	< 2e-16
COVID-19 cases per 100k	covariate	0.00024
COVID-19 deaths per 100k	covariate	0.030
Prior COVID-19 test positivity	covariate	0.91
COVID-19 vaccination	covariate	< 2e-16
Hospitalization days or ER/urgent care visits in previous month	covariate	1.49e-08

[1] – p-values without adjustment for multiple hypothesis testing
