# Supplementary material for: Factors associated with anxiety during the first two years of the COVID-19 pandemic in the United States: An analysis of the COVID-19 Citizen Science study
Source: PLoS One. 2024 Feb 6;19(2):e0297922. doi: 10.1371/journal.pone.0297922 (PMC10846720; doi:10.1371/journal.pone.0297922)
Supplement: S6 Table — (PDF) [file pone.0297922.s007.pdf]

S6 Table. Associations between COVID-19 health worry, difficulty paying for basic living expenses, and anxiety during the COVID-19 pandemic using the anonymized S1 dataset.

COVID-19 Health Worry	GAD-7 Score difference (95% confidence intervals)[1]
	**Fully adjusted model**[2]
Not worried or other	0 (reference)
A little worried	0.36 (0.33, 0.39)
Somewhat worried	0.94 (0.92, 0.97)
Very worried	1.87 (1.84, 1.91)
Extremely worried	3.26 (3.22, 3.31)
p-value for trend	p < 2e-16
adjusted R^2	0.25
**Difficulty Paying for Basics**[3]	
**Not very hard**	0 (reference)
**Somewhat hard**	0.69 (0.65, 0.72)
**Hard**	1.41 (1.33, 1.49)
**Very hard**	2.10 (2.00, 2.20)
p-value for trend	p < 2e-16
adjusted R^2	0.25

[1] – Estimates and 95% confidence intervals represent the estimated difference in the Generalized Anxiety Disorder (GAD-7) score (scale 0-21) compared to the indicated reference category from multivariable linear regression models. p < 2e-16 for all point estimates shown.

[2] – Adjusted similarly to Model 3 of Table 3 in the main text for the following covariates: days elapsed following declaration of COVID-19 as a global pandemic by the WHO on March 11, 2020, repeated measures from individual participants, baseline demographic characteristics, medical conditions, subjective social status, time-varying COVID-19 case and death rates (for the county containing the largest proportion of residences associated with each participant's zip code), personal COVID-19 experience and vaccination status, and the other independent variable of interest (both Economic Stress and COVID-19 Worry were included in the model). No interaction terms are included.

[3] – "Prefer not to state" and "Don't know" categories for economic stress are omitted from the table.

GAD-7 - Generalized Anxiety Disorder (GAD-7) questionnaire
